# Gut-Pancreas-Liver Axis as a Target for Treatment of NAFLD/NASH

**DOI:** 10.3390/ijms21165820

**Published:** 2020-08-13

**Authors:** Gianluca Svegliati-Baroni, Bárbara Patrício, Gessica Lioci, Maria Paula Macedo, Amalia Gastaldelli

**Affiliations:** 1 Liver Injury and Transplant Unit, Ospedali Riuniti Ancona, 60020 Ancona, Italy; gsvegliati@gmail.com; 2 Obesity Center, Polytechnic University of Marche, 60121 Ancona, Italy; 3 Cardiometabolic Risk Unit, Institute of Clinical Physiology, CNR, 56124 Pisa, Italy; barbara_patricio@ifc.cnr.it; 4 Institute of Life Sciences, Sant′Anna School of Advanced Studies, 56127 Pisa, Italy; 5 APDP Diabetes Portugal, Education and Research Center (APDP-ERC), 1250-189 Lisbon, Portugal; 6 Department of Gastroenterology, Polytechnic University of Marche, 60121 Ancona, Italy; gessicalioci@gmail.com; 7 CEDOC, NOVA Medical School/Faculdade de Ciências Médicas, Universidade Nova de Lisboa, 1150-082 Lisboa, Portugal; 8 Department of Medical Sciences, Institute of Biomedicine-iBiMED, University of Aveiro, 3810-193 Aveiro, Portugal

**Keywords:** non-alcoholic fatty liver disease, non-alcoholic steatohepatitis, type-2 diabetes, gut-pancreas-liver axis, incretins, lipid metabolism, glucose metabolism

## Abstract

Non-alcoholic fatty liver disease (NAFLD) represents the most common form of chronic liver disease worldwide. Due to its association with obesity and diabetes and the fall in hepatitis C virus morbidity, cirrhosis in NAFLD is becoming the most frequent indication to liver transplantation, but the pathogenetic mechanisms are still not completely understood. The so-called gut-liver axis has gained enormous interest when data showed that its alteration can lead to NAFLD development and might favor the occurrence of non-alcoholic steatohepatitis (NASH). Moreover, several therapeutic approaches targeting the gut-pancreas-liver axis, e.g., incretins, showed promising results in NASH treatment. In this review, we describe the role of incretin hormones in NAFLD/NASH pathogenesis and treatment and how metagenomic/metabolomic alterations in the gut microbiota can lead to NASH in the presence of gut barrier modifications favoring the passage of bacteria or bacterial products in the portal circulation, i.e., bacterial translocation.

## 1. Introduction

The incidence of non-alcoholic fatty liver disease (NAFLD) is rapidly growing and it is estimated that in the general population about 25% of the individuals have hepatic steatosis [1]. The real prevalence of the disease is unknown, but it increases up to 57% in those with body mass index (BMI) of 30kg/m^2^ or greater (obesity), to 70% in diabetic subjects, and 90% in morbidly obese patients undergoing metabolic surgery [2,3].

The mechanisms that lead to hepatic triglyceride (TG) accumulation and the development of NASH are complex and still incompletely understood. It has been postulated that the initial accumulation of hepatic TG is a compensatory effect for hepatic lipid overflow without major metabolic effect (first hit). However, lipotoxicity and other factors are triggering hepatic inflammation and tissue fibrosis that can lead to non-alcoholic steatohepatitis (NASH, second hit) [4]. Several hypotheses have been postulated as regard to the second hit, including the involvement of an alteration in the gut-liver axis [5]. The intestine is a multifunctional organ connected with many body organs but its role in metabolism control was only revealed in recent years. The intestinal cells secrete several hormones—among these glucagon-like peptide-1 (GLP-1), glucose-dependent insulinotropic peptide (GIP), oxyntomodulin and possibly glucagon—that are involved with pancreatic hormones insulin and glucagon and are important for the maintenance of glucose and lipid homeostasis [6]. Moreover, the gut microbiota can influence whole body and liver metabolic and inflammatory status through the metabolism of nutrients and the release of anti- and pro-inflammatory compounds. The intestinal barrier might become compromised allowing bacterial translocation from the intestine to other organs (the liver in primis). The aim of this review is to discuss some of the mechanisms involved in the gut-pancreas-liver axis important for the development of NAFLD and its progression to NASH and other comorbidities like type-2 diabetes (T2D).

## 2. Physiological Effects of Incretin Hormones

**Figure 1:**
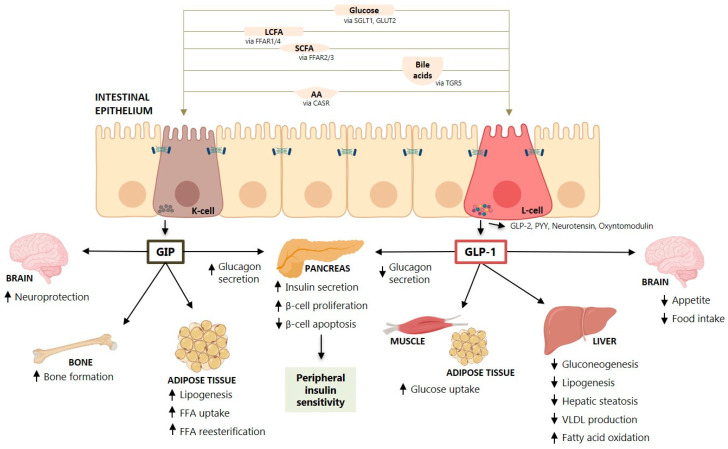
Secretion and physiological effects of incretin hormones GIP and GLP-1 in different organs and tissues. The intestinal epithelium is composed of several cell types, including enteroendocrine cells such as K-cells and L-cells, responsible for the secretion of GIP and GLP-1, respectively. The secretion of these hormones is triggered upon nutrient stimulation of G protein-coupled receptors (GPCRs) present in the cell membrane. Some of the nutrients responsible for GIP and GLP-1 secretion are glucose (and other carbohydrates), lipids, some amino acids and proteins. Bile acids can also stimulate incretin secretion. After secretion, GIP and GLP-1 exert their functions in the pancreas and extra-pancreatic tissues, such as the liver, adipose tissue, muscle, bone, and the central nervous system. Abbreviations: AA, Amino acids; CASR, Calcium-sensing receptor; FFAR, Free fatty acid receptor; GIP, Glucose-dependent insulinotropic peptide; GLP-1, Glucagon-like peptide-1; GLP-2, Glucagon-like peptide-2; GLUT, Glucose transporter; LCFA, Long-chain fatty acids; PYY, Peptide YY; SCFA, Short-chain fatty acids; SGLT, Sodium-dependent glucose cotransporter; TGR5, Takeda G protein-coupled receptor 5; VLDL, Very-low-density lipoprotein.

It is now recognized that the intestine is an endocrine organ able to secrete hormones that regulate whole-body metabolism in response to food ingestion and also to regulate appetite and gastric emptying. Among these hormones, the most studied are the incretin hormones like GLP-1 and GIP that are secreted mainly by the intestine in response to a meal [7] (Figure 1).

After secretion, gut hormones are rapidly degraded by dipeptidyl peptidase-4 (DPP-4), resulting in an in vivo half-life of approximately two minutes. DPP-4 is a 110 kilodaltons (kDa) membrane-associated peptidase that is expressed on the apical surface of epithelial and acinar cells, endothelial cells, fibroblasts, and lymphocytes [8,9,10] and also exists as a soluble circulating form in plasma [11]. For this reason, synthetic forms of GLP-1 resistant to the action of DPP-4 (exenatide, liraglutide, lixisenatide, albiglutide, dulaglutide, semaglutide) or inhibitors of DPP-4 activity (e.g., gliptins) have been developed and commercialized in order to raise the plasma levels of GLP-1.

### 2.1. Incretin Secretion and Physiological Effects

The secretion of the incretin hormones GLP-1 and GIP occurs in the intestine in response to a meal [7] (Figure 1). Nutrients in a meal, such as glucose, fatty acids and triglycerides, amino acids and proteins stimulate incretin hormone secretion in the intestine [12]. Some studies showed that pancreatic alpha cells may also secrete GLP-1 [13]. The major effect of incretins is the potentiation of glucose-stimulated insulin secretion by pancreatic β-cells [14,15,16,17]. Despite similar effects on insulin secretion, GLP-1 and GIP have opposite effects on glucagon secretion by pancreatic α cells. While GLP-1 suppresses glucagon secretion at euglycemia, but not during hypoglycemia [18], GIP stimulates glucagon secretion at euglycemia, but not during hyperglycemia [19], being one of the reasons behind the apathy for GIP-based therapies for T2D. The early studies by Nauck and Holst have shown that the actions of GLP-1 and GIP on insulin secretion are nearly equal and additive in healthy humans [20,21], which is not observed in the set of T2D where the endocrine pancreas ceases responsiveness to GIP but remains responsive to GLP-1, explaining the reduced/absent incretin effect of GIP in T2D [22,23].

GLP-1 also has other pleiotropic effects (Figure 1), including delay in gastric emptying and glucose absorption [24,25], stimulation of brain areas involved in the regulation of glucose homeostasis and food reward [26], suppression of glucagon secretion [25], beneficial effects on cardiovascular risk factors, a natriuretic effect on the kidney and others [16,27]. Of importance, GLP-1 has been shown to have a central effect to promote satiety, to reduce appetite and food intake [28], and GLP-1 receptor agonists (GLP-1RA) have been shown to promote weight loss and decrease liver fat [28,29] (Figure 1). GIP is also an incretin hormone but its power to stimulate insulin secretion was considered minor compared to GLP-1 [20]. A recent study showed that GIP’s effects on glucose excursions and insulin secretion in response to a four-hour oral glucose tolerance tests (75 g) combined with an ad libitum meal test were more relevant than GLP-1′s effects (i.e., higher postprandial plasma glucose excursions and lower insulin secretion) and that the two hormones contribute additively to postprandial glucose regulation in healthy individuals [30]. Secretion of these two hormones is altered by obesity, age, diabetes, weight loss and exercise [31,32,33]. However, the pleiotropic effects of GLP-1 and GIP seem to be directed to different organs (Figure 1), such as the adipose tissue, muscle, central nervous system, bone and the liver.

### 2.2. Incretin Effects on Liver Enzymes, Steatosis, Inflammation and Fibrosis

The long-term effects of GLP-1RA go beyond the improvement in glucose metabolism and weight loss [34,35,36,37,38,39] and it is now established their beneficial effects of hepatic metabolism and histology. Only a limited number of studies have evaluated in humans the impact of GLP-1RA or DPP-4 inhibitors on liver function and hepatic steatosis (Table 1 and Table 2). In general, a reduction in hepatic TG content has been observed with GLP-1RA. All GLP-1RA have shown an improvement in liver enzymes that was more evident in subjects with elevated alanine aminotransferase (ALT) at baseline.

The effect of GLP-1RA on hepatic steatosis was observed in several mice models (including leptin-deficient ob/ob mice, leptin receptor-deficient db/db mice, and high-fat diet (HFD) fed mice that showed a decrease after exenatide treatment [160,161,162] or liraglutide [163].

Liraglutide treatment reduced ALT in a dose-dependent matter (although the difference was significant only at the maximum dose, i.e., at 1.8 mg) [34,164]. Post-hoc analyses on the effects of other GLP-1RA on liver enzymes showed a reduction in liver enzymes with lixisenatide [113] and exenatide [110,144,153]. Among the long-acting GLP-1RA, both dulaglutide [106,107] and semaglutide treatments were associated with improvement in ALT more evident in subjects with elevated ALT before treatment and in a dose-dependent way. Liraglutide promoted weight loss and beneficially altered the fat distribution by decreasing waist circumference, waist/hip ratio, and the amount of subcutaneous and visceral fat content and liver steatosis [145,150,152,155]. An ancillary study of the LEAD-2 (Liraglutide Effect and Action in Diabetes) trial analyzed the changes in liver, visceral and subcutaneous fat by computed tomography as liver-to-spleen attenuation ratio in 131 subjects treated with various doses of liraglutide+metformin versus placebo or glimepiride [145]. Liraglutide decreased liver fat only in the 1.8mg/day+metformin group while changes in 0.6 and 1.2 mg/day liraglutide, placebo+metformin or glimepiride+metformin were not significantly different from baseline [145]. The LEAN (Liraglutide Efficacy and Action in Non-Alcoholic Steatohepatitis) trial is the largest trial so far that evaluates the efficacy of a GLP-1RA (i.e., liraglutide) in biopsy-proven NASH patients. Fifty-two patients were randomly assigned 1:1 to subcutaneous injections of liraglutide (1.8 mg daily) or placebo for 48 weeks [103]. Liraglutide induced resolution of NASH in 39% of patients versus 9% of placebo.

Treatment with exenatide reduced liver steatosis [109,146,150] and when added to pioglitazone, an oral hypoglycemic and insulin sensitizing drug approved for the treatment of T2D that acts on nuclear peroxisome proliferator activated receptor-gamma (PPAR-γ) [147], potentiated the known action of pioglitazone in reducing hepatic fat [92]. Similar effects were observed in the Duration-8 trial where exenatide alone or in combination with SGLT2 inhibitor dapagliflozin significantly reduced the liver fat scores [111].

Studies in animal models indicate that GLP-1RA treatment might be beneficial also for liver fibrosis. However, conclusive data on the effects of GLP-1RA on hepatic fibrosis and parameters of liver disease in humans are lacking (Table 2). In the LEAN trial fewer patients in the liraglutide group (2/23) showed progression in fibrosis compared to the placebo group (8/22), and a greater proportion of liraglutide-treated patients improved steatosis and hepatocyte ballooning, while no differences were seen in lobular inflammation and overall NAFLD activity score [103]. Trevaskis et al. showed that liraglutide treatment reduced immunostaining for type I collagen, the most abundant type in hepatic fibrosis [165]. In a retrospective study, liraglutide and pioglitazone, but not sitagliptin, decreased liver fibrosis index APRI (calculated as the aspartate aminotransferase (AST) to platelet count ratio), and the improvement was associated with weight loss and a marked reduction in AST and ALT levels [149]. An open-labeled, prospective study examined the effect of exenatide in eight T2D patients with biopsy-proven NAFLD [146]. After 28 weeks, liver histology assessed using the NAFLD Activity Score improved in three of eight subjects and fibrosis was reduced in four of eight subjects [146]. However, the lack of a control group and the small number of subjects is a major limitation in this study. In the study by Eguchi, 10 patients that received over two-year treatment with liraglutide showed improved histology while only 1 subject worsened his condition [155].

The number of patients enrolled in studies with GLP-1RA was often limited, without a control group. However, the meta-analysis performed by Dong et al. that included six studies (three with liraglutide and three with exenatide, but only three with liver biopsy including the LEAN study) showed that treatment with GLP-1RA reduced, compared to placebo, the degree of NASH activity (in terms of steatosis, lobular inflammation and hepatocytes ballooning) and the stage of fibrosis [166].

### 2.3. Effect of GLP-1 and GLP-1RA on Hepatic Glucose and Lipid Metabolism

**Figure 2:**
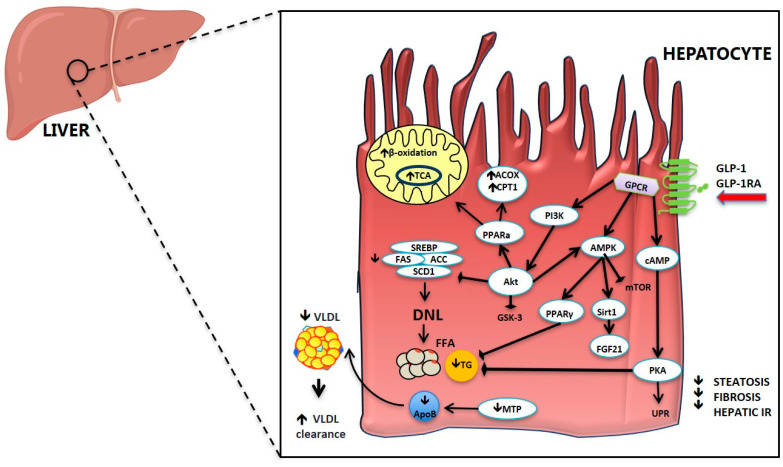
Mechanistic effects of GLP-1 and GLP-1 receptor agonists in hepatic lipid metabolism. GLP-1 and GLP-1 receptor agonists act via G protein-coupled receptors (GPCRs) in hepatocytes to target several signaling pathways, resulting in increased β-oxidation in mitochondria, decreased hepatic mRNA expression of several lipogenic genes (SREBP1, FAS, ACC and SCD1), reduced de novo lipogenesis (DNL) and reduced steatosis. Abbreviations: ACC, Acetyl-CoA carboxylase α; ACOX, Peroxisomal acyl-coenzyme A oxidase; Akt, Serine/threonine protein kinase B/Akt; AMPK, 5′ AMP-activated protein kinase; ApoB, Apolipoprotein B 100; cAMP, Cyclic adenosine monophosphate; CPT1, Carnitine palmitoyltransferase 1; DNL, de novo lipogenesis; FAS, Fatty acid synthase; FFA, Free fatty acids; FGF21, Fibroblast growth factor 21; GLP-1, Glucagon-like peptide-1; GLP-1RA, Glucagon-like peptide-1 receptor agonists; GPCR, G-protein-coupled receptors; GSK-3, Glycogen synthase kinase 3; MTP, Microsomal triglyceride transfer protein; mTOR, mammalian target of rapamycin; PI3K, Phosphoinositide 3-kinase; PKA, Protein kinase A; PPAR, Peroxisome proliferator-activated receptor; SCD1, Stearoyl-CoA desaturase 1; Sirt1, Sirtuin 1; SREBP, Sterol regulatory element-binding protein; TCA, Tricarboxylic acid cycle; TG, Triglyceride; UPR, Unfolded protein response; VLDL, Very-low-density lipoprotein.

The liver has a crucial role in maintaining glucose homeostasis, which is tightly orchestrated by the action of pancreatic insulin and glucagon. Gut hormones, particularly incretins, are also involved in this regulation (Figure 2) not only through the potentiation of insulin secretion. Several studies have shown an improvement in hepatic glucose and lipid metabolism after GLP-1 injection [167,168] or GLP-1RA treatment [24,102,169,170,171] either in humans and animal studies.

The positive effect of GLP-1RA on diabetic hyperglycemia and hepatic metabolism appears to be due in part to weight loss through their effects on appetite and food consumption and in part to reduced glucotoxicity in T2D. However, infusion of native GLP-1 showed acute effects on glucose metabolism by reducing hepatic glucose production [167,168].

GLP-1 receptor was found in nerves that innervate hepatocytes [172], but their presence on human liver or hepatocytes is debated as found by some [169,170] but not others [173,174]. A recent study that used incretins (GLP-1 and Exendin-4) radiolabeled with technetium-99m showed that the tracer was highly taken up both by the liver and the pancreas and that the uptake was reduced in both organs after diet-induced obesity (DIO), similarly to alloxan diabetes controls [175].

In addition to the glucose-lowering actions, GLP-1RA based therapies have been reported to improve both fasting and postprandial lipid profiles in patients with T2D, in particular triglyceride concentrations [104,147,153,176,177]. Native GLP-1 did not change free fatty acid (FFA) concentrations or glycerol rate of appearance [168]. On the other hand, peripheral lipolysis is often found reduced after GLP-1RA treatment [102,178] possibly because of weight loss. However, it is now evident that GLP-1RA improves the antilipolytic effect of insulin by reducing adipocyte insulin resistance and the overflow of FFA to the liver [24,102].

The mechanism(s) through which gut hormones regulate hepatic lipid metabolism are still unclear. In Figure 2 we have summarized some of the proposed mechanisms for GLP-1 and GLP-1RA. In vitro studies have demonstrated a direct effect of GLP-1 and GLP-1RA on hepatocyte fatty acid metabolism [102,170,179]. It has been proposed that GLP-1RA ameliorate hepatic steatosis by increasing fatty acid oxidation, enhancing very-low-density lipoprotein (VLDL) secretion and inhibiting lipogenesis [170,180]. Treatment of Huh7 and HepG2 hepatoma cells with exenatide decreased intracellular lipid accumulation [181,182] by enhancing fatty acid oxidation and reducing lipogenesis [160,161,179,183]. In hepatocytes from rats that developed NASH after HFD, exenatide activated genes involved in hepatic fatty acid β-oxidation and insulin sensitivity such as PPAR-γ expression, protein kinase A activity, serine/threonine protein kinase B/Akt and AMP-activated protein kinase (AMPK) phosphorylation, and PPAR-α activity, that were altered by HFD treatment [170] (Figure 2). GLP-1-treated hepatocytes manifested a significant increase in cAMP production and a reduction in mRNA expression of stearoyl-CoA desaturase 1 and genes associated with fatty acid synthesis, such as acetyl-CoA carboxylase and fatty acid synthase [160,184]. The converse was true for genes associated with fatty acid oxidation [160].

GLP-1RA treatment has been associated with alterations in lipid and lipoprotein metabolism in patients with T2D. Administration of a single subcutaneous injection of exenatide (10μg) prior to a meal was shown to reduce postprandial TG, apolipoproteins B-48 and CIII, remnant lipoprotein (RLP)-cholesterol and RLP-TG in comparison to saline injection [185]. Other studies reported similar results with liraglutide once-daily for three weeks [186] and 16 weeks [104]. Whyte et al. showed that a four-week treatment with lixisenatide reduced chylomicron (CM)-TG pool-size and [^13^C] oleate concentrations, and increased CM-TG clearance without affecting CM-TG production rate. These data lead the authors to conclude that the decreased CM-TG concentration may result from increased clearance rather than decreased production of lipoproteins [187]. Additionally, a four-week treatment with DPP-4 inhibitor vildagliptin was also found to induce reductions in the postprandial TG and apolipoprotein B-48 containing TG-rich lipoprotein particle metabolism after a fat meal [177].

### 2.4. Impact of DPP-4 and Its Inhibition on NAFLD

The dipeptidyl peptidase-4 (DPP-4), also known as CD26, is a ubiquitously expressed protease that degrades several substrates, such as incretin hormones and thereby is a therapeutic target in diabetes treatment. DPP-4 is highly expressed in liver and DPP-4 mRNA expression is increased in the liver of patients with NAFLD, compared to healthy subjects [188]. Moreover, serum DPP-4 activity and hepatic staining of DPP-4 are correlated with NASH grade but do not correlate with the fasting blood glucose concentration or glycosylated hemoglobin (HbA1c) [189,190]. These observations suggest that hepatic DPP-4 expression in NAFLD might be directly associated with hepatic lipogenesis and liver injury but not with alteration in glucose metabolism. 

Few data are available with DPP-4 inhibitors and NAFLD/NASH (Table 2). Studies in mice reported a reduction in liver TG and histologic grade of hepatic steatosis with sitagliptin [183] but if this is a direct or indirect effect is uncertain. DPP-4 deficient rats have three-fold higher basal GLP-1 levels and significantly reduced serum and hepatic TG level compared with controls [179]. Patients with NASH have increased serum DPP-4 activity compared to controls and also increased hepatic staining of DPP-4 that correlated with hepatic steatosis and histopathological grade of NASH but were not correlated to each other [190]. In a small cohort of T2D patients with NASH, one-year treatment with the DPP-4 inhibitor sitagliptin decreased hepatocyte ballooning and improved NASH score [148]. The improvement in hepatic histology was correlated to the reduction in BMI and serum AST and ALT levels. However, the reduction in steatosis score was of borderline statistical significance and no change was observed in either liver fibrosis or lobular inflammation [115,148] (Table 2). The available data suggest that treatment with DPP-4 inhibitors may have a mild beneficial effect on NAFLD, probably mediated by the increase in GLP-1 concentrations after blocking DPP-4 activity.

It is important to note that DPP-4, also known as CD26, can also derive from the gut, due to the microbiota DPP-4-like activity [191]. Recently, reduction of DPP-4 activity by vildagliptin was linked with the prevention of gut permeability disruption in mice subjected to western diet [191]. Moreover, Olivares et al. suggested that this effect is in part due to the vildagliptin properties on the microbiota-derived DPP-4. Even though DPP-4/CD26 is expressed at high levels in the intestine, specifically in the jejunum and ileum, its role in promoting gut permeability is still unclear [192].

### 2.5. GLP-1 Co-Agonists in the Treatment of Lipid Disorders and NAFLD/NASH

Several drugs that combine GLP-1RA and other hormones—e.g., combined double co-agonism of GLP-1 receptors and glucagon, an analogue of oxyntomodulin, combination GLP-1/GIP or triple co-agonism of the of GLP-1/GIP/glucagon receptors—are in the pipeline for the treatment of T2D and obesity, and have been studied in the context of NAFLD/NASH [193,194,195] (Table 1 and Table 3). GIP targets the white adipose tissue (Figure 1) improving its buffering capacity by promoting postprandial triglyceride storage and fatty acids re-esterification [196]. Glucagon has also effects on lipid metabolism stimulates lipolysis and hepatic fatty acid oxidation [197] but its effects should be always be considered relative to the insulin levels since insulin exerts opposite effects on lipid metabolism [198].

Once per week administration of a dual GLP-1/glucagon co-agonist attached to polyethylene glycol (PEG), a process called PEGylation, improved glucose tolerance, reduced body weight and body fat by decreasing food intake and increasing energy expenditure, while normalizing liver lipid content, within a four-week treatment in DIO mice [202]. Day et al. further optimized the co-agonism to equally align activity at each receptor, which maximized weight-loss and glucose-lowering effects [203]. Another study in DIO mice injected with a protease-resistant dual GLP-1/glucagon co-agonist showed similar results in terms of weight loss, lipid-lowering activity, anti-hyperglycemic efficacy, increased fatty acid oxidation and reduced hepatic steatosis [204]. In methionine/choline-deficient diet-fed mice, two-week treatment with a dual GLP-1/glucagon co-agonist (G49) ameliorated NASH by reducing inflammation, steatosis, oxidative stress, and apoptosis and increased mitochondrial biogenesis [205]. Interestingly, PEGylated dual GLP-1/glucagon co-agonism was also shown to restore leptin sensitivity in DIO mice continuously exposed to HFD, and the combination with PEGylated leptin resulted in a greater weight loss and improvement in lipid and glucose metabolism when compared with PEGylated GLP-1/glucagon alone [206]. In HFD-induced NAFLD mice, treatment with a dual GLP-1/glucagon co-agonist for 40 weeks prevented body weight gain, glucose intolerance and insulin resistance, while it decreased circulating and hepatic lipid content. Histological assessment of these animals showed a reduced fat accumulation, inflammation and fibrosis in co-agonist treated animals [207]. In comparison with liraglutide alone, MEDI0382, a dual GLP-1/glucagon co-agonist achieved greater weight loss and comparable glucose-lowering actions in DIO mice and non-human primates [208]. In patients with T2D, the phase 2a study of once-daily subcutaneous injections of MEDI0382 (up to 200µg) for 41 days significantly decreased glucose area under the curve after four hours of a mixed-meal tolerance test, and body weight in comparison with placebo [64]. In another trial, SAR425899, a dual GLP-1/glucagon co-agonist, significantly reduced fasting plasma glucose, HbA1c, and body weight in patients with T2D as well as in healthy volunteers when compared to placebo [199].

Another class of drugs are the dual GIP/GLP-1 co-agonists **(**Table 1 and Table 3). Once-weekly administration of tirzepatide (LY3298176), a dual GIP/GLP-1 co-agonist, for 26 weeks significantly reduced HbA1c and body weight in a dose-dependent way, and at the highest dose (15 mg) reduced total cholesterol and TG concentrations in comparison both with placebo and dulaglutide 1.5 mg in patients with T2D [84]. More recently, Hartman et al. found that once-weekly administration of tirzepatide for 26 weeks significantly reduced biomarkers of NASH and fibrosis (ALT, AST, keratin-18, and procollagen-C3) and increased adiponectin compared with placebo at a higher tirzepatide dose (15 mg) in patients with T2D [85].

The fatty-acylated dual GLP-1/GIP co-agonist NNC0090-2746, also known as NN9709, MAR709, and RG7697, significantly improved glycemic control and reduced body weight and total cholesterol compared with placebo after a 12-week phase 2 trial [87]. Similarly, treatment with RG7697 for 14 days yielded a reduction in fasting and postprandial plasma glucose in a dose-dependent way in patients with T2D in comparison with placebo, as well as a decrease in postprandial insulin, suggesting an improvement in insulin sensitivity [209].

A triple agonist of GLP-1, GIP and glucagon receptors (Table 1 and Table 3) was found to have better results in terms of reduction of body weight, improvement in glycemic control and reversion of hepatic steatosis in rodents in comparison with existing dual co-agonists and best-in-class mono-agonists [210]. The authors showed that the metabolic efficacy of the triple agonist results from the synergistic action of glucagon to increase energy expenditure, GLP-1 to reduce food intake and improve glucose homeostasis, and GIP to potentiate incretin role and protect against the glucagon diabetogenic effect [210]. In body fat mass matched male and female mice, treatment with GLP-1/GIP/glucagon triple agonism inhibited food intake, improved dyslipidemia, and decreased body weight and body fat mass in both groups, and reversed diet-induced steatohepatitis to a larger extent in female mice compared to male mice [211]. Therefore, development of therapies based on dual and triple co-agonists of GLP-1, GIP and glucagon receptors might be of interest (Table 3), not only for T2D or obesity but also for NAFLD/NASH; however, more studies are needed.

## 3. Gut Microbiota Dysbiosis and the Gut-Liver Axis in NAFLD

**Figure 3:**
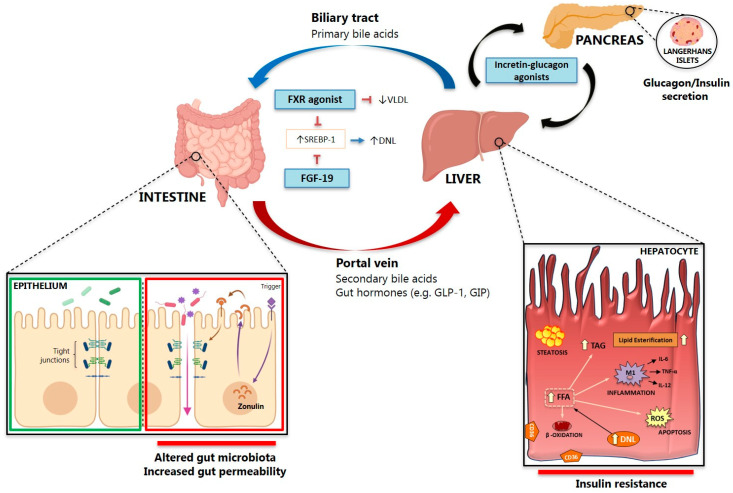
The gut-pancreas-liver axis in the context of non-alcoholic fatty liver disease (NAFLD). The intestine and the liver are in constant communication through the portal vein and the biliary tract. In the intestine, the maintenance of the intestinal endothelial cell barrier achieved through tight junctions is crucial. In pathological conditions, an alteration in the diversity and composition of gut microbiota could lead to the secretion of harmful bacterial products, which consequently damages the intestinal epithelium by disrupting tight junctions. Another mechanism that has been indicated to explain increased intestinal permeability is zonulin, a protein secreted within intestinal cells upon a trigger (e.g., gluten), which is thought to be responsible for tight junction disassembling. In such conditions, harmful bacteria/bacterial products cross the intestinal wall and reach the liver via portal circulation, where they activate toll-like receptors (TLRs) on hepatocyte cell surface, promoting steatosis, inflammation, apoptosis, and insulin resistance. The intestine is also responsible for the secretion of the incretin hormones GLP-1 and GIP upon nutrient stimulation, which go through the liver, reach circulation, and target the pancreas to regulate insulin and glucagon secretion. On the other hand, the liver secretes primary bile acids into the intestine to aid digestion, which are converted into secondary bile acids by gut microbiota, and the majority are reabsorbed back to the liver. A rearrangement in the gut-pancreas-liver axis has been hypothesized to play a role in the pathogenesis and development of NAFLD. Abbreviations: CD36, Cluster of differentiation 36; DNL, de novo lipogenesis; FFA, Free fatty acids; GIP, Glucose-dependent insulinotropic peptide; GLP-1, Glucagon-like peptide-1; IL, Interleukin; ROS, Reactive oxygen species; TAG, Triglycerides; TNF-α, Tumor necrosis factor α.

The intestine is anatomically wired to the liver and brain through blood circulation and systemic innervation. In this perspective, intestinal dysfunction has been investigated as a determinant of hepatic impairment. A large body of evidence collected in recent years supports the novel concept that the gut-liver axis wires physiological mechanisms of inter-organ control affected in many metabolic diseases. The gut is colonized by a wide range of microorganisms, including bacteria that co-evolved with the host in a symbiotic relationship – the gut microbiota. The gut microbiota includes a diverse range of symbiotic bacteria and other microorganisms that colonize the human intestine [212]. These bacteria produce a large array of metabolites that serve as important signaling factors and energy substrates, such as bile acids (BA), choline, neurotransmitters, and short-chain fatty acids (SCFA) [213]. The crosstalk between these metabolites and the host modulates the immune system, regulates metabolic phenotypes, and influences risk factors for disease and response to therapy (Figure 3). The gut microbiota composition differs along the intestinal tract depending on physiological conditions, such as gastrointestinal content flow rates, availability of substrates for microorganisms, lumen pH, oxygen availability and immunological features [214]. For example, a large number of species and diversity are lowered in the small intestine compared to the colon, since the small intestine is characterized by adverse conditions for bacterial growth, such as low pH, antimicrobials response and short transit time; in this tract, the most abundant species are *Lactobacillaceae* and *Enterobacteriaceae* [215]. At the phylum level, gut microbiota composition is dominated by Firmicutes and *Bacteroidetes*, followed by *Proteobacteria* and *Verrucomicrobia* [216].

“Normal” gut flora maintains a symbiotic relationship with the host, which confers benefits to both species in terms of immune system development and efficiency and energy metabolism regulation [217]. However, when qualitative and quantitative modifications of the gut microbiota occur, i.e., dysbiosis, the development of chronic diseases, including obesity, diabetes and NAFLD is promoted [218]. The association between dysbiosis and these metabolic conditions was firstly suggested by Turnbaugh et al. [219] where the authors showed that transferring gut microbiota from obese to lean rodents induced in the recipient the same metabolic alterations observed in the donor. 

From then, it has been shown that dysbiosis can influence the occurrence of progression of NAFLD through several mechanisms. The gut microbiomes of patients with advanced liver disease and cirrhosis are characterized by an increase in potentially pathogenic bacteria, along with reduced numbers of bacteria with beneficial properties [220,221,222,223,224,225]. Mouzaki et al. found that patients with NASH have a lower fecal percentage of *Bacteroidetes* compared to both patients with simple hepatic steatosis and healthy controls [224]. Zhu et al. have found in pediatric patients that *Proteobacteria*, *Enterobacteriaceae*, and *Escherichia* were the only phylum, family and genus types exhibiting a significant difference between obese and NASH microbiomes. They also found that *Proteobacteria* produced significant ethanol levels that could be responsible for the higher degree of liver injury [225].

In NAFLD models, lack of inflammasomes has been demonstrated to select a pathogenetic microbiota which was associated with a more severe degree of liver injury [226]. In addition, it has been recently shown that a Western lifestyle diet associated with lack of NLRP3-inflammasome, increased fat storage in adipose tissue and liver leading to a more severe liver injury. This effect was associated with the expansion of Gram-negative *Proteobacteria* and *Verrucomicrobia* [227], the main pathobionts responsible of liver injury in a model of HFD and fibrosis [228], and together with *Verrucomicrobia* are known to be mucus-degrading bacteria, favoring bacterial translocation. In patients with hepatocellular carcinoma (HCC), *Bacteroides* and *Ruminococcaceae* were increased, while Bifidobacterium was reduced, and *Akkermansia* and *Bifidobacterium* were inversely correlated with calprotectin, a marker of intestinal inflammation that was increased in HCC patients [229]. 

In addition to its role in modulating the progression of chronic liver disease, differences in microbiota composition have been attempted to stratify NAFLD patients by degree of liver injury. Boursier et al. found that NAFLD severity was associated with gut dysbiosis and a shift in the metabolic function of the gut microbiota with *Bacteroides* independently associated with the occurrence of NASH, and *Ruminococcus* with the presence of significant fibrosis [230]. Del Chierico et al. [231] showed that either in obese or in NAFLD/NASH pediatric patients, microbiota diversity was reduced from controls to obese, NAFLD and NASH patients, and the combination of specific microbiota and metabolite modifications was able to discriminate control patients from those with NAFLD or NASH [231]. Similarly, Loomba et al. constructed a metagenomic-based model to distinguish advanced fibrosis in adult patients with NASH based on increased *Proteobacteria* (mostly *E. Coli*) levels and a decrease in *Firmicutes* [232]. Studies performed in cohorts of morbidly obese women confirmed the increased in Gram- proteobacteria and linked, for the first time, steatosis occurrence with alterations in aromatic and branched-chain amino acid metabolism [233].

### Alterations of Lipid and Glucose Metabolism by Gut Bacteria

Bacterial biodiversity has been associated with a most favorable metabolic profile, but bacterial products can, directly and indirectly, influence glucose and lipid metabolism. Changes in synthesis and production of SCFA by colonic bacterial fermentation of polysaccharides (such as acetate, propionate, and butyrate) are the most studied mechanisms relating the association between gut microbiota and development/progression of NAFLD. A close correlation between SCFA and NAFLD development have been demonstrated both in experimental models of NAFLD and obese patients that have increased concentration of stool SCFA [219,234]. SCFA can bind specifically to GPCRs such as GPR41 and GPR43, which are expressed in the adipose tissue, liver, and intestine and induce metabolic modifications influencing de novo lipogenesis, cholesterol synthesis and glucose homeostasis [235]. Increased acetate in the liver can cause TG accumulation as acetate is an important substrate for fatty acid synthesis, while increased levels of propionate promote gluconeogenesis in the liver since propionate is a precursor for gluconeogenesis [236].

Moreover, SCFA can stimulate the release of GLP-1 in vitro and in vivo dependently of the FFA receptor 2 (Figure 1) [237]. Furthermore, SCFA can modulate food intake by regulating neuronal activity and thus appetite regulation [219]. Although SCFA directly or via binding to G-protein-coupled receptors (GPCR) appear to promote NAFLD, other effects of SCFA might be beneficial for NAFLD mostly by modulating AMPK activity in the liver [236]. Furthermore, butyrate has been demonstrated to influence colonic epithelium regeneration, thus providing an important mechanism in the regulation of gut integrity [238]. Propionate can also bind to GPCR-43 expressed on lymphocytes to maintain appropriate immune defense. Butyrate activates PPAR-γ leading to beta-oxidation and oxygen consumption, a phenomenon contributing to maintaining anaerobic condition in the gut lumen [239].

A second mechanism relates to the direct effect on lipid metabolism. One of the most known effects is the capacity of intestinal bacteria to inhibit the synthesis of fasting-induced adipocyte factor (also known as angiopoietin-related protein 4), a specific inhibitor of the lipoprotein lipase. Lack of inhibition of lipoprotein lipase leads to a stronger release (spillover) of FFA from VLDL particles to the liver, favoring the development of steatosis [240]. Finally, the host response to translocated microbial products predicts outcomes also in patients with hepatitis B and C virus infections, indicating that bacterial translocation contributes to the progression of chronic liver injury not only in the presence of metabolic or alcoholic disease [241].

## 4. Bile Acid Signaling in NAFLD

One of the most intriguing aspects of the role of dysbiosis in the progression of chronic liver injury is related to the interactions between gut microbiota and bile acids (BA). In hepatocytes, BA are synthesized via cytochrome P450 (CYP)-mediated oxidation of cholesterol by two biosynthetic pathways: the “classical” and “alternative” pathways. The “classical” pathway produces the primary BA cholic acid and chenodeoxycholic acid through the enzymatic actions of cholesterol hydroxylase enzymes CYP7A1, CYP8B1, and CYP27A1. The “alternative” pathway yields chenodeoxycholic acid via the hydroxylation of the cholesterol sidechain by CYP27A1, followed by 7α-hydroxylation of 27-hydroxycholesterol and other oxysterols by CYP7B1. Following the conjugation of cholic acid and chenodeoxycholic acid to either taurine or glycine to form taurocholic acid, glycocholic acid, taurochenodeoxycholic acid (TCDCA) and glycochenodeoxycholic acid (GCDCA), primary BA are secreted from the liver into the bile canaliculus via the canalicular bile salt export pump [242]. 

In the gut, gut bacteria metabolize BA, and glyco-conjugated and tauro-conjugated cholic and chenodeoxycholic acids are deconjugated via bile salt hydrolases and 7α-dehydroxylated to form secondary BA deoxycholic acid (DCA) and lithocholic acid (LCA) [243]. The ability of the gut microbiota to metabolize intestinal BA into their unconjugated forms is central to metabolic homeostasis since these unconjugated BA can activate BA signaling receptors such as farnesoid X receptor (FXR), pregnane X receptor, constitutive androstane receptor, vitamin D receptor and Takeda G protein receptor 5 (TGR5) [242].

Normal intestinal FXR activity maintains an efflux of BA back into the portal vein and a controlled reuptake of BA into enterocytes. This limits intracellular BA levels and regulates the expression of fibroblast growth factor (FGF)-15 in mice and its orthologue FGF19 in humans, which inhibit BA synthesis in hepatocytes via the activation of hepatic FGF receptor 4 [243]. During intestinal inflammation, the expression of FXR is downregulated leading to an increased influx of BA into the enterocytes and reduced BA efflux back into the portal vein, with the net effect of higher BA levels in the intestinal mucosa. Thus, intestinal FXR downregulation diminishes signaling via the FGF19–FGF receptor 4 axis leading to (a) increased BA synthesis in the liver; (b) increased hepatic influx of BA; (c) detoxification and BA biliary secretion blockage. These series of events induce cholestasis followed by alterations in carbohydrate and lipid metabolisms, and induces inflammation, leading to the upregulation and activation of factor nuclear kappa B (NF-κB), which then binds directly to the FXR promoter to inhibit its transcription [244]. An example of the complex interactions between diet, dysbiosis and BA comes from the observation that coupling an HFD with a chemical carcinogen to induce a shift to a Gram-positive microbiota was able to increase DCA levels, to induce a senescence-associated secretory phenotype in hepatic stellate cells that activated the inflammasome system, leading to increased HCC formation. This was confirmed with a non-selective antibiotic cocktail that induced tumor reduction and by the use of the antibiotic vancomycin that selectively acts on Gram-positive bacteria [245].

The secondary BA DCA is known to promote DNA damage and hepatic carcinoma and accumulates significantly on an animal product-based diet in humans [246]. Increased circulating DCA levels have been found in patients with NASH [247]. Neonatal mice mimicking all the histological spectrum of NAFLD that were treated with streptozocin to induce diabetes and HFD-fed showed increased lipopolysaccharide (LPS) levels both in the liver and in feces, increased hepatic BA levels, and down-regulation of the FXR/FGF15 axis. The progression of chronic liver injury was associated with a relationship between dysbiosis and specific BA formation (DCA, LCA, TCDCA, GCDCA) with an increase in specific bacteria genera (Clostridium, Bacteroides, E. Coli) and reduction in *A. muciniphila* that significantly correlated with specific increases in secondary BA [248,249]. These BA (DCA, TCDCA, GCDCA) are significantly increased in patients with NASH [247].

### Targeting BA Receptors as a Therapeutic Option for NAFLD 

FXR agonism has shown benefit in several preclinical models of NAFLD/NASH, inducing metabolic effects that reduce steatosis and inflammation (Figure 3) [250]. On the other hand, a direct effect of FXR agonism on hepatic fibrosis is controversial [251]. This has led to the design of clinical trials involving the FXR agonist obeticholic acid (OCA) in human patients (Table 1). In the first trial, Mudaliar et al. found improved insulin resistance and decreased liver fibrosis markers in diabetic patients with NAFLD treated with OCA [82]. Then, the FLINT trial (FXR Ligand Obeticholic Acid in NASH Treatment, NCT01265498) included NASH patients treated with OCA (25 mg/day) for 72 weeks [83]. Although patients treated with OCA showed a significant improvement in the NAFLD activity score (i.e., the primary histological outcome) and a significant decrease in liver fibrosis, NASH resolution occurred in only 22% of patients treated with OCA (without a statically significant effect compared to placebo), and no effect was observed in patients with advanced fibrosis. Although OCA was generally well tolerated, concerns were raised by the occurrence of side effects such as pruritus, which developed in 23% of patients on OCA without leading to drug discontinuation, increased total serum cholesterol and low-density lipoprotein (LDL)-cholesterol levels and a modest, but significant, reduction in high-density lipoprotein (HDL)-cholesterol [83]. More recently, the interim analysis at month 18 of the phase 3 trial REGENERATE showed that 23% and 18% of the subjects in the 25 mg and 10 mg group respectively vs 12% in the placebo group met the primary endpoint, i.e., fibrosis improvement by at least one stage with no worsening of NASH with a similar adverse event and safety profile compared to the phase 2 trial [81]. However, these results were not sufficient to receive FAD accelerated approval for the treatment of patients with liver fibrosis due to NASH [252].

The side effect on cholesterol levels might be important when considering that patients with NAFLD/NASH have an increased risk of cardiovascular diseases and high cholesterol is a major risk factor for atherosclerosis. Thus, further studies are needed to better define the clinical usefulness of FXR agonism in NASH. Firstly, the site of FXR agonism can be of critical importance. Moreover, while mice with whole-body FXR deficiency spontaneously develop liver tumors [253], De Girolamo et al. showed that no tumor formation was observed in mice expressing FXR only at the intestine level [254]. The gut-restricted FXR agonist fexaramine robustly induces enteric FGF15, leading to alterations in BA composition, without activating FXR target genes in the liver and reducing diet-induced weight gain, body-wide inflammation, hepatic glucose production, while enhancing thermogenesis and browning of white adipose tissue [255]. Conversely, some data also indicate that antagonism of intestinal FXR signaling could improve metabolic parameters in murine models of NAFLD [256,257]. The underlying mechanisms are still unclear but may be related to the existence of an “intestinal FXR/ceramide axis” whereby ileal production of ceramides, mediated by BA modification by the microbiota, controls the hepatic lipogenic pathway via sterol regulatory element-binding protein 1c (SRBEP1c).

Finally, concerns can be raised regarding the chronically increased levels of FGF19 resulting from FXR agonism. FGF19 has the potential to regulate several pathways involved in NASH pathogenesis since it can act on CYP7A1, the rate limiting enzyme in BA synthesis from cholesterol, in addition to mimicking the effect of insulin on glycogen synthesis and gluconeogenesis [258]. However, the therapeutic potential of FGF19 has been hindered by its effect on potential occurrence of HCC, since mice expressing an FGF19 transgene developed liver tumors while tumor FGF19 hepatic mRNA expression was an independent prognostic factor for overall and disease-free survival [259,260]. It has been found that the tumorigenic effect of FGF19 is mediated by the release of interleukin 6 (IL6) by Kupffer cells that activate the signal transducer and activator of transcription (STAT3) pathway in hepatocytes [261]. On the other hand, an engineered FGF19 analogue (NGM82) has shown efficacy in animal models of NASH. Furthermore, NGM282 can block hepatocarcinogenesis associated with human FGF19. In animal models of NASH, treatment with NGM282 resulted in the reduction of ALT and AST, as well as improvement in all histological features associated with NASH—hepatic steatosis, inflammation, ballooning degeneration, and fibrosis—thus leading to test NGM282 in clinical trials [262,263,264] (Table 1). In the first clinical trial, NGM282 showed significant reductions in liver fat content, as measured by magnetic resonance imaging proton density fat fraction, with an acceptable safety profile in patients with NASH [59]. Following this, in a recent open-label study, NGM282 improved the histological features of NASH with significant reductions in NAS and fibrosis scores, accompanied by improvements in noninvasive imaging and serum markers, although this effect was observed after only 12 weeks of treatment [60].

## 5. Gut Permeability

The term intestinal permeability refers to the intestinal epithelium as a barrier that physiologically controls the exchange of ions, water and macromolecules between the luminal content and the underlying tissues, and that is protected from direct contact with bacteria, food allergens, toxins, and excreted metabolic byproducts by a mucus layer. The apical surface of the epithelium is a continuum of single cells whose paracellular space is sealed by a series of intercellular junctions, of which tight junctions (TJ) (Figure 3). One type of TJ is involved in the high-capacity pore pathway to transport ions and small molecules, and the other type is involved in the leak pathway, modulating the flux of large macromolecules [265]. Proteins of the TJ such as claudins play a relevant role in the high capacity pore pathway, whereas zonula occludens-1 (ZO-1 or Tjp1), occludin, and tricellulin (also known as Marveld2) control the low-capacity leak pathway [265].

Zonulin, the only physiological modulator of tight junctions described to date, was discovered as the precursor of haptoglobin-2 (pre-Hp2) [266] and is essential for the regulation of intestinal permeability [267,268,269] (Figure 3). Zonulin is the only measurable blood protein that reflects intestinal permeability, and increased zonulin levels are considered to be a marker of impaired intestinal barrier [269]. The epithelial barrier dysfunction may be triggered by the presence of some microbial communities and their derived metabolites [270,271], which can be assessed by circulating zonulin as well as by circulating LPS. Additionally, intestinal permeability can also be assessed by orally administered inert or metabolic active probes, which can be differentially transported through the intestinal epithelium and detected in the blood or urine. For example, lactulose/mannitol ratio in urine is informative on detecting the small intestine permeability, while increased levels of sucrose or sucralose excretion are associated with large intestine permeability [272]. However, the development of a unique gold standard and complete evaluation of this phenomenon are needed, since only 39% of NAFLD patients and 49% of NASH patients have demonstrated intestinal permeability [273].

Increased intestinal permeability has been found in several conditions including exposure to HFD, specific microbiota, gliadin, allergens or even hyperglycemia [227,269]. Thaiss et al. clearly showed that hyperglycemia drives intestinal permeability by the glucose transporter GLUT2-dependent transcriptional reprogramming of intestinal epithelial cells, resulting in changes in tight and adherence junction integrity, and a systemic influx of microbial products into portal blood [274]. Moreover, the inflammasome, a multiprotein complex that orchestrates host defense mechanisms against infectious agents, appears to be also linked to intestinal permeability. HFD impinged intestinal permeability which was more pronounced in the NLRP3-inflammasome deficient mice in a ZO-1 dependent manner [227]. This phenomenon was associated with changes in diet-induced anti-microbial peptides secretion and with an inflammasome-dependent downregulation of angiogenin-4, an intestinal protein with important antibacterial functions [227]. The authors proposed that the combination of a Western lifestyle diet with inflammasome alteration modified antimicrobial activity, which might lead to a boosted abundance of mucus degrading bacteria, allowing passage of pathogens into the portal circulation [227]. Another mechanism involved in the regulation of intestinal barrier function is the COX-2/prostaglandin E2 (PGE2)/EP-2/p-ERK integrated signal pathways that modulate the expression of intestinal ZO-1 and cell adhesion molecule E-cadherin [275]. Gao J et al. observed that the restore of the COX-2/prostaglandin E2 (PGE2)/EP-2/p-ERK pathway using celecoxib in a thioacetamide rat model improved intestinal permeability by blocking the inflammatory response, as shown by decreased levels of LPS, tumor necrosis factor (TNF)-α and interleukin (IL)-6, thus reducing the progression of liver injury to cirrhosis [275].

### 5.1. Gut Permeability and Bacterial Translocations

Bacterial translocation can be defined as the passage of bacteria, or bacterial products such as LPS, into the portal circulation and happens when the complex regulation of the mechanisms of the gut barrier is lost [276], which has been linked with the occurrence of NAFLD, NASH, hepatic fibrosis, and HCC in several rodent models [227,228,277]. The association between increased gut permeability and NAFLD has also been demonstrated in human studies, and bacterial translocation occurs in those patients that develop NASH, at least in a pediatric study [278]. In NASH patients, serum LPS and hepatocytes LPS localization was higher than controls, which was correlated with serum zonulin and pNF-κB expression [279]. Recently it has been shown that disruption of the intestinal epithelial barrier and gut vascular barrier are early events in NASH pathogenesis and that OCA is able to protect against barrier disruption and thereby prevents the development of NASH, providing further evidence for its use in the prevention or treatment of NASH [280].

Specifically, bacterial components can increase the expression of specific receptors, such as Toll-Like Receptors (TLRs). TLRs can bind PAPMs (small molecular motifs conserved within a class of microbes) and DAMPs (host biomolecules that can initiate and perpetuate a noninfectious inflammatory response) leading to increased gene expression of pro-inflammatory mediators such as TNFα, IL-1β, and interferons. In NASH, TLR4 (that recognizes LPS, a gram-negative bacteria wall component), TLR5 (known to recognize bacterial flagellin from invading mobile bacteria), TLR2 (that recognizes many bacterial, fungal, viral, and endogenous substances) and TLR9 (a specific receptor for double-stranded bacterial DNA) have been shown to play a key pathogenetic role [281]. Increased levels of LPS activate TLR4 and have been observed in the serum of steatotic patients and in diet-induced NAFLD/NASH models (HFD, fructose-rich diet, methionine/choline-deficient diet, and choline-deficient L-amino acid-defined diet), where lack of TLR4 showed reduced liver damage, in terms of steatosis or steatohepatitis [279,282]. Similar results indicating a pathogenetic role of TLR have been obtained also for TLR9 using the choline-deficient L-amino acid-defined diet model of NASH [283]. Furthermore, FAs also have been recognized as TLR4 ligands that can activate pro-inflammatory signaling leading to TNFα secretion [284]. In a HFD plus fructose model of NASH, liver injury development was associated with dysbiosis, bacterial translocation mediated by alterations in intestinal permeability due to reduced tight junctions’ expression, and reduced gut antimicrobial capacity. These results indicate that complex interactions between the diet, the innate immunity, bacteria and intestinal function occurs in the development of NASH [226,227], and similar observations were obtained in experimental models of HCC development [285,286,287].

TLRs signaling can also act via the activation of inflammasomes. Once activated, the inflammasomes trigger an intracellular cascade which results in the secretion of pro-inflammatory and pro-fibrotic IL-1β and IL-18. Either in patients with NASH or animal models, the expression of inflammasome components was increased, indicating the potential role in the pathogenesis of liver injury [226,227,288,289,290,291]. The mechanisms behind the effects of the inflammasomes in NAFLD are complex and might involve metabolic, inflammatory and pro-fibrogenic effect in the liver (either parenchymal and in non-parenchymal cells), adipose tissue, and immune system. In the last few years, the role of the inflammasome in the modification of the gut microbiota have been highlighted. The inflammasome can indeed strongly affect gut microbiota composition as deletion of inflammasome components was associated with loss of epithelial integrity and gut inflammation associated with dysbiosis and bacteremia [292].

In comparison with healthy controls, patients with NASH are more likely to present an increased intestinal permeability. Luther et al. [273] also showed that an initial stage of hepatic damage and inflammation is associated with an alteration in intestinal permeability. Moreover, intestinal permeability and the prevalence of small intestinal bacterial overgrowth are correlated with the severity of liver steatosis [293]. The same pattern is seen in obese subjects with steatosis who present an increase in intestinal permeability in comparison with obese patients [294]. Patients with NAFLD ranging from simple NAFLD to NASH showed higher intestinal permeability as assessed by a lactulose/mannitol test, alcohol, and endotoxin levels in plasma in comparison with controls [295]. It has been also shown that individuals with celiac disease, characterized by increased gut permeability, are at increased risk of NAFLD compared to the general population [296,297].

### 5.2. Gut-Derived Inflammatory Mediators Triggering NAFLD/NASH

The increase in gut permeability allows a constant flow of pro-inflammatory mediators to the liver, which firstly target the liver resident macrophages, the Kupffer cells. Several compounds bind to TLRs expressed in Kupffer cells and also in hepatic stellate cells, namely methylamine, volatile organic compounds and pathogen-associated molecular patterns, which include LPS, lipoteichoic acid, peptidoglycan, flagellin and unmethylated CpG DNA, and activate inflammatory signaling pathways like the NF-kB [298]. Kupffer cell activation results in a subclinical hepatic inflammatory state that may lead to the development of NAFLD/NASH. Of relevance among TLRs, TLR4 identifies LPS from gram-negative bacteria and TLR9 recognizes bacteria-derived GpG-containing DNA. Hence, TLR4 knockout mice show protection towards the manifestation of liver injury. Even though Kupffer cells contain the highest expression of TLR4, stellate cells TLR4 signaling appear to be critical in transforming growth factor β secretion and fibrosis development in a myeloid differentiation primary response 88 (MyD88) dependent manner [299]. Kupffer cells are not only sensitive to inflammatory signals but also to metabolic challenges such as dyslipidemia, which affects Kupffer cell activation in models of liver steatosis and steatohepatitis [300,301]. Another molecule that has been implicated in liver fibrosis resolution is DPP-4/CD26, whose increased circulating activity is inversely correlated with a drastic reduction in Kupffer cell population upon liver injury [302]. Latest studies strongly suggest that Western diet-fed mice under inflamed gut conditions driven by dextran sulfate sodium 1% exacerbate hepatic steatosis, inflammation and fibrosis progression with increased IL-1, IL-6, TNF-α, monocyte chemoattractant protein (MCP)-1 and profibrogenic factors, such as transforming growth factor (TGF)-β, α2-Actin, tissue inhibitor of metalloproteinase (TIMP)-1 and plasminogen activator inhibitor (PAI)-1 [303].

## 6. Targeting the Gut Permeability to Improve NAFLD/NASH

Two types of strategies can be used to improve gut permeability to target NAFLD/NASH pathogenesis and progression. The first one is to address gut microbiota in a way that gut inflammation is rescued, and permeability is restored. The second strategy is to target gut inflammation/permeability itself but there are still a few therapeutic strategies nowadays. 

For the first strategy, a pharmacological approach to simultaneously impact on the microbiota and the gut permeability and ultimately target the liver can be applied. Biguanides/metformin, the first-line therapeutics for T2D, modifies microbiota leading to an increase in *A. muciniphila*, as well as several SCFA-producing microbiota, favoring an improvement on mucin-producing goblet cells, pro-inflammatory IL-6, and glucose tolerance [304]. A direct effect of metformin on the ileal epithelial barrier was also observed and the authors associated this improvement to increased activation of AMPK [305]. In another study, metformin improved tight junction integrity and permeability by increasing occludin-1 levels and increasing *A. muciniphila* levels [306]. Thus, metformin not only directly improved NAFLD but also affected the epithelial intestinal barrier.

Similarly, DPP-4 inhibitors are also able to modify gut microbiota by increasing *Firmicutes* and *Tenericutes* phyla and decreasing *Bacteroidetes* in DIO rodents [307]. Nonetheless, vildagliptin reduces *Firmicutes* to *Bacteroidetes* ratio, increases *Lactobacilli spp*. and increases propionate production [308]. Moreover, Olivares et al. suggested that this vildagliptin effect is in part due to its properties on the microbiota-derived DPP-4 [191]. Regarding GLP-1RA, scarce literature is available. However, liraglutide appears to enhance SCFA-producing bacteria including *Bacteroides*, *Lachnospiraceae* and probiotic bacteria, *Bifidobacterium*, and the authors attribute the change in local inflammation to this gut microbiota structural modifications [309]. Whether individuals that are more responsive to GLP-1 secretion have a gut microbiota more shielded towards NAFLD and dysmetabolism is unveiled. The SGLT2 inhibitor dapagliflozin also showed some improvement in NAFLD, affecting microbiota by promoting a reduction of the *Firmicutes*/*Bacteroidetes* ratio in diabetic mice [310]. As previously described, disruption of the intestinal epithelial barrier and gut vascular barrier represents early events in NASH pathogenesis and are mediated by the occurrence of dysbiosis. OCA repairs these impairments through its FXR-dependent effect [280]. It has been already demonstrated that intestinal FXR modifies the gut microbiota and activates the TGR5/GLP-1 axis and this lead to improvement of hepatic glucose and insulin sensitivity and increase adipose tissue browning [311]. These studies reinforce the hypothesis of a direct link between intestinal nuclear receptors and gut microbiota in modifying the gut-liver axis function in the host metabolic control.

More recently, a few papers have evaluated the role of *A. Muciniphila* [312,313,314]. In humans, studies have provided evidence for a negative correlation between *A. Muciniphila* abundance and overweight, obesity, untreated T2D or hypertension. In mice, it has been shown that administration of *A. Muciniphila* prevents the development of obesity and associated complications. However, the sensitivity of *A. Muciniphila* to oxygen limited the development of translational approaches for human medicine. However, it was found that both pasteurization of *A. Muciniphila* and a specific protein isolated from its outer membrane (Amuc_1100) recapitulates the beneficial effect of the entire bacterium facilitating the possibility of its use in clinical trial in humans. In a proof-of-concept study, administration of live or pasteurized *A. Muciniphila* improved insulin sensitivity and reduced insulinemia and plasma total cholesterol without affecting body weight, fat mass and hip circumference. 

Moreover, the beneficial effects of probiotics in the modulation of gut microbiota to achieve metabolic improvements in the host are minimal [315]. In a preliminary report, human-to-human fecal microbial transplantation has demonstrated a beneficial influence of the microbiota from a lean donor in obese recipients, resulting in improved insulin sensitivity [316]. 

However, no further data are available on direct manipulation of gut microbiota. Several signs of progress have been made on knowledge of NASH pathogenesis, but several aspects still need to be clarified and they are mostly related to the gut-liver axis mechanisms. This is the reason why there is nowadays a lack of effective therapies directly targeting the gut-microbiota-liver axis for the treatment of NASH and its progression to hepatic cirrhosis and end-stage liver disease.

## 7. Conclusions

Currently, available data reviewed herein suggest a role for GLP-1RA in ameliorating hepatic insulin resistance and improving NAFLD with beneficial effects on hepatic lipid metabolism. Given their beneficial effects on liver enzymes, weight loss and hepatic metabolism, GLP-1RA (currently approved for the treatment of diabetic hyperglycemia and obesity) may prove to be an effective treatment for NAFLD/NASH in nondiabetic subjects, as well as individuals with diabetes. Because DPP-4 inhibitors do not increase (only prolong) the elevation of circulating GLP-1 levels nor induce major weight loss, it is unclear whether they will have a significant beneficial effect on the prevention/treatment of NASH/NAFLD, as opposed to the GLP-1RA which are administered in pharmacologic amounts. Further studies are required to assess their efficacy, mechanism of action, and safety of GLP-1RA and DPP-4 inhibitors in patients with NAFLD/NASH. Other drugs with known beneficial effects on NASH (pioglitazone) or with significant effects in phase 2 (GLP-1RA) or 3 (FXR agonists) trial indicate the key role of gut-liver axis alterations in promoting the progression of NAFLD to NASH. Although alterations in gut microbiota have been associated with obesity and NAFLD there is no evidence indicating that in humans a long-term change in gut bacterial composition can be achieved and improves NAFLD/NASH. A better knowledge of the mechanisms leading to increased intestinal permeability and small metabolites produced during dysbiosis will provide novel therapeutic targets to prevent the development and progression of NAFLD to NASH.

## Figures and Tables

**Table 1:** Drugs with known effect on parameters of NAFLD/NASH (divided by the clinical trial phase).

Drug Name	Mechanism of Action	Admini-Stration	Stage of Development	Effect on Steatosis	Effect on Fibrosis	Effect on LFTs	Effect on Glucose Metabolism	Effect on Insulin Resistance	References
**HM15211**	GLP-1/GIP/glucagon	SC	Phase I	Yes *	Yes *	Yes *	NA	NA	[40,41,42]
**NGM313 MK3655**	Activator of FGFR1c/KL	SC (once monthly)	Phase I	Yes	NA	NA	Yes	Yes	[43,44]
**RO5093151**	11β-HSD1 inhibitor	PO	Phase I/II	Yes	NA	Yes	Yes	Yes	[45,46]
**MK-0533**	PPAR γ/SPPARM	PO	Phase II	NA	NA	NA	Yes	Yes	[47]
**Pemafibrate**	PPAR α/SPPARM	PO	Phase II	NA	NA	Yes	NA	Yes	[48]
**Saroglitazar**	PPAR α/γ	PO	Phase IIa	Yes	Yes	Yes	Yes	Yes	[49,50,51]
**Lanifibranor**	PPAR α/γ/δ	PO	Phase IIa	Yes	Yes	Yes	Yes	Yes	[52,53]
**Tropifexor**	FXR agonist	PO	Phase IIb	Yes	Yes	Yes	NA	NA	[54,55]
**Cilofexor**	FXR agonist	PO	Phase II	Yes	No	Yes	NA	NA	[56,57]
**NGM282**	FGF19 analogue	SC	Phase II	Yes	Yes	Yes	No	No	[58,59,60,61,62,63]
**Cotadutide (MEDI0382)**	GLP-1/glucagon	SC	Phase II	Yes	NA	Yes	Yes	Yes	[64,65]
**ZP2929/BI 456906**	GLP-1/glucagon	SC	Phase II	Yes *	Yes *	NA	Yes *	Yes	[66,67]
**Licogliflozin**	Dual SGLT1/2 inhibitor	PO	Phase IIa	Yes	NA	Yes	Yes	Related to weight loss	[68,69]
**BIO89-100**	PEG-FGF21 analogue	SC	Phase Ib/IIa	Yes	NA	Yes	Yes	Yes	[70]
**PF-06835919**	Ketohexokinase (KHK) Inhibitor	PO	Phase II	Yes	NA	Yes	NA	Yes	[71]
**TVB-2640**	Fatty acid synthase (FAS) inhibitor	PO	Phase II	Yes	NA	Yes	No	No	[72]
**GS-0976 (Firsocostat)**	Acetyl-CoA carboxylase (ACC) inhibitor	PO	Phase II	Yes	Yes	Yes	No	No	[73]
**Resmetirom (MGL-3196)**	Hepatic thyroid hormone receptor-β agonist	PO	Phase II/III	Yes	Yes	Yes	NA	NA	[74]
**MSDC-0602K**	Mitochondrial pyruvate carrier (MPC)	PO	Phase IIb	Yes	NA	Yes	Yes	Yes	[75,76]
**Lobeglitazone**	PPAR γ	PO	Phase III	Yes	No	NA	NA	Yes	[77]
**INT-131 besylate**	PPAR γ/SPPARM	PO	Phase III	Yes	No	NA	Yes	Yes	[78]
**Elafibranor**	PPAR α/δ	PO	Phase III	Yes	Yes	Yes	Yes	Yes	[79,80]
**Obeticholic acid**	FXR agonist	PO	Phase III	Yes, mild	Yes	Yes	No	NA	[81,82,83]
**Tirzepatide (LY3298176)**	GLP-1/GIP	SC	Phase III	Yes	Yes	Yes	Yes	Yes	[84,85,86]
**NNC0090-2746/RG7697**	GLP-1/GIP	SC	Phase III	Yes	Yes	Yes	Yes	Yes	[87]
**Pegbelfermin**	Long-acting FGF21 analogue	SC	Phase III	Yes	Yes	Yes	No	No	[88,89]
**Aramchol**	Stearoyl-coenzyme-A-desaturase-1 (SCD1) inhibitor	PO	Phase III/IV	Yes	Yes	Yes	Yes	No	[90,91]
**Pioglitazone**	PPAR γ	PO	Phase IV	Marked	Yes	Yes	Yes	Yes	[92,93,94,95]
**Fenofibrate**	PPAR α	PO	Phase IV	Minimal	No	Yes	No	No	[96,97,98]
**Bezafibrate**	PPAR α	PO	Phase IV	NA	NA	Yes	No	No	[99,100,101]
**Liraglutide**	GLP-1RA	SC	Phase IV	Yes	Yes	Yes	Yes	Related to weight loss	[102,103,104]
**Semaglutide**	GLP-1RA	SC/PO	Phase IV	Yes	Yes	Yes	Yes	Related to weight loss	[105]
**Dulaglutide**	GLP-1RA	SC	Phase IV	Yes	Yes	Yes	Yes	Related to weight loss	[106,107]
**Exenatide**	GLP-1RA	SC	Phase IV	Yes	Yes	Yes	Yes	Related to weight loss	[24,108,109,110,111,112]
**Lixisenatide**	GLP-1RA	SC	Phase IV	NA	NA	Yes	Yes	Related to weight loss	[113]
**Sitagliptin**	DPP-4 inhibitor	PO	Phase IV	No	No	No	Yes	No	[114,115,116,117]
**Empagliflozin**	SGLT2 inhibitor	PO	Phase IV	Yes	Yes	Yes	Yes	Related to weight loss	[118,119,120]
**Canagliflozin**	SGLT2 inhibitor	PO	Phase IV	Yes	NA	Yes	Yes	Related to weight loss	[121,122,123,124,125,126]
**Dapagliflozin**	SGLT2 inhibitor	PO	Phase IV	Yes	NA	Yes	Yes	Related to weight loss	[111,127,128,129,130]
**Ertugliflozin**	SGLT2 inhibitor	PO	Phase IV	NA	NA	Yes	Yes	Related to weight loss	[131]
**Ipragliflozin**	SGLT2 inhibitor	PO	Approved by PMDA	Yes	Tendency to decrease	Yes	Yes	Related to weight loss	[117,132,133,134,135,136,137,138]
**Tofogliflozin**	SGLT2 inhibitor	PO	Approved by PMDA	Yes	NA	Yes	Yes	Related to weight loss	[139]
**Luseogliflozin**	SGLT2 inhibitor	PO	Approved by PMDA	Yes	NA	Yes	Yes	Related to weight loss	[140,141]
**Glargine**	Insulin	SC	Phase IV	Yes	NA	No	Yes	-	[112,142,143]

Only drugs approved or under investigation in clinical trials were reported. Abbreviations: ACC, Acetyl-CoA carboxylase α; DPP-4, Dipeptidyl peptidase 4; FAS, Fatty acid synthase; FGF, Fibroblast growth factor; FGFR1c/KLB: fibroblast growth factor receptor 1c/βKlotho complex; FXR, Farnesoid X receptor; GIP, Glucose-dependent insulinotropic peptide; GLP-1, Glucagon-like peptide-1; GLP-1RA, Glucagon-like peptide-1 receptor agonists; HSD1, Hydroxysteroid dehydrogenase; KHK, Ketohexokinase; MPC, Mitochondrial pyruvate carrier; NA, Not available; PEG, PEGylated molecule attached to polyethylene glycol (PEG); PMDA, Pharmaceuticals and Medical Devices Agency; PO, Oral administration; PPAR, Peroxisome proliferator-activated receptor; SC, Subcutaneous administration; SCD1, Stearoyl-CoA desaturase 1; SGLT, Sodium-dependent glucose cotransporter; SPPARM, Selective PPARγ modulator. * Any changes in histological parameters were published only for preclinical studies (especially for the studies in phase 1).

**Table 2:** Liver-related outcome effects of treatment with GLP-1RA or DPP-4 inhibitors in humans.

References	Subjects	Weeks of Treatment	Treatment Agent	Histology (Yes/No)	Fibrosis	Steatosis	Visceral Fat	ALT	BMI	HbA1c
Buse et al. (2007) [144]	T2D (*n* = 283)	30 + 2 years follow up	Exenatide	No	-	-	-	↓ (if elevated at baseline)	↓	↓
Klonoff et al. (2008) [110]	T2D (*n* = 217)	30 + 3 years follow up	Exenatide	No	-	-	-	↓ (if elevated at baseline)	↓	↓
Jendle et al. (2009) [145]	T2D (*n* = 131)	26	Liraglutide + Metformin	No	-	↓ (CT)	↓ (CT)	↓	↓	↓
Kenny et al. (2010) [146]	T2D and biopsy-proven NAFLD (*n* = 8)	28	Exenatide	Yes	↓	↓	-	↓	↓	↓
Sathyanarayana et al. (2011) [147]	T2D (*n* = 21)	52	Exenatide + Pioglitazone	No	-	↓ (MRS)	-	↓	↓	↓
Yilmaz et al. (2012) [148]	T2D+ NASH (*n* = 15)	52	Sitagliptin	Yes	=	=	-	↓	↓	=
Ohki et al. (2012) [149]	T2D and NAFLD (*n* = 26)	NA	Liraglutide	No	↓ (APRI)	-	-	↓	↓	↓
Ohki et al. (2012) [149]	T2D and NAFLD (*n* = 36)	NA	Sitagliptin	No	= (APRI)	-	-	↓	=	↓
Cuthberson et al. (2012) [150]	Obese, T2D and NAFLD (*n* = 25, 19/6)	26	Exenatide or Liraglutide	No	-	↓ (MRS)	↓ (MRI)	↓	↓	↓
Fan et al. (2013) [151]	T2D and NAFLD (*n* = 117)	12	Exenatide or Metformin	No	-	-	-	↓	↓	=
Suzuki et al. (2013) [152]	T2D (*n* = 46)	26	Liraglutide + Pioglitazone	No	-	↓ (CT)	↓ (CT)	-	↓	=
Bergenstal et al. (2013) [153]	T2D (*n* = 534)	52	Exenatide	No	-	-	-	↓	↓	↓
Blaslov et al. (2014) [154]	T2D (*n* = 125)	26	Exenatide alone or add-on metformin or/and sulphonylurea	No	-	↓ (FLI)	-	↓	↓	↓
Shao et al. (2014) [109]	Obese, NAFLD with elevated liver enzymes and T2D (*n* = 60)	12	Exenatide + Insulin	No	-	↓ (US)	-	↓	↓	↓
Watanabe et al. (2015) [115]	T2D and NAFLD (*n* = 7)	12	Sitagliptin	No	-	↓ (MRS)	= (MRI)	=	=	↓
Tang et al. (2015) [142]	T2D (*n* = 35)	12	Liraglutide or Insulin glargine	No	-	= (MRS)	-	=	↓	↓
Eguchi et al.* (2015) [155]	Diabetic NAFLD/NASH (*n* = 19)	24 + 24	Lifestyle + Liraglutide	No	↓ (FIB-4)	↓ (CT)	↓ (CT)	↓	↓	↓
Eguchi et al.* (2015) [155]	T2D+ NAFLD/NASH (*n* = 10)	24 + 24 + 96	Lifestyle + Liraglutide + Follow-up Liraglutide	Yes	↓	↓	-	↓	↓	↓
Smits et al. (2016) [156]	Overweight T2D (*n* = 52)	12	Liraglutide or Sitagliptin	No	= (APRI/FIB-4/NFS)	= (MRS)	-	↓	=	↓
Cui et al. (2016) [114]	Prediabetic NAFLD (*n* = 50)	24	Sitagliptin	No	= (MRE)	= (MRI)	-	=	=	=
Armstrong et al. (2016) [103]	Biopsy-proven NASH (*n* = 23)	48	Liraglutide	Yes	↓	↓	-	↓	↓	↓
Joy et al. (2017) [116]	Biopsy-proven NASH (*n* = 12)	26	Sitagliptin	Yes	= (NAS)	= (MRI)	= (MRI)	=	=	↓
Petit et al. (2017) [157]	T2D (*n* = 68)	26	Liraglutide	No	-	↓ (MRS)	-	↓	↓	↓
Feng et al. (2017) [158]	T2D and NAFLD (*n* = 87)	24	Liraglutide, Metformin or Gliclazide	No	-	↓ (US)	-	↓	↓	↓
Khoo et al. (2017) [159]	Obese NAFLD (*n* = 24)	26	Liraglutide versus Lifestyle	No	-	↓ (MRI)	-	↓	↓	-
Seko et al. (2017) [107]	T2D and biopsy-proven NAFLD (*n* = 15)	12	Dulaglutide	Yes	↓	↓	-	↓	↓	↓
Cusi et al. (2018) [106]	T2D (*n* = 971)	26	Dulaglutide	No	-	-	-	↓	-	↓
Yan et al. (2019) [143]	T2D and NAFLD (*n* = 18)	26	Liraglutide + Metformin	No	-	↓ (MRI)	↓ (MRI)	-	↓	↓
Gastaldelli et al. (2019) [111]	T2D (*n* = 228)	28/52	Exenatide + Dapagliflozin	No	-	-	-	↓	-	↓

Abbreviations: ALT, alanine aminotransferase; APRI, AST to platelet ratio index; BMI, body mass index; CT, computed tomography; FIB-4, fibrosis-4 index; HbA1c, glycosylated hemoglobin; MRE, magnetic resonance elastography; MRI, magnetic resonance imaging; MRS, magnetic resonance spectroscopy; NAFLD, non-alcoholic fatty liver disease; NAS, NAFLD Activity Score; NASH, Non-alcoholic steatohepatitis; NFS, NAFLD fibrosis score; T2D, type-2 diabetes; US, ultrasound. ↓ represent a decrease; = represent no change. * 24weeks lifestyle intervention, followed by 24 weeks with liraglutide. 10 subjects continued liraglutide until 96 weeks and were followed up with biopsy. All analyses are reported versus baseline GLP-1RA/DPP-4 inhibitor.

**Table 3:** Current GLP-1, GIP, and glucagon receptor co-agonists in development for treatment of T2D/obesity.

Co-agonism	Drug	Company	Stage of Development	References
GLP-1/glucagon	Cotadutide (MEDI0382)	Medimmune	Phase II	[64,65]
	HM12525A; JNJ-64565111	Hanmi Pharmaceuticals	Phase II	[67]
	MK-8521	Merck	Phase II	[67]
	SAR425899	Sanofi	Phase II	[199]
	ZP2929; BI 456906	Zealand Pharma	Phase II	[66,67]
	JNJ-54728518	Janssen Pharmaceuticals	Phase I	[200]
	NN9277; NNC 9204-1177	Novo Nordisk	Phase I	-
	MOD-6030	Prolor/OPKO Biologics	Phase I	[201]
	VPD-107	Spitfire Pharma	Pre-clinical	-
GLP-1/GIP	Tirzepatide (LY3298176)	Eli Lilly	Phase III	[84,85,86]
	NNC0090-2746; NN970 9; MAR709; RG7697	Novo Nordisk/Marcadia	Phase III-stopped	[87]
	CPD86	Eli Lilly	Pre-clinical	-
	ZP-I-98	Zealand Pharma	Pre-clinical	-
	ZP-DI-70	Zealand Pharma	Pre-clinical	-
GLP-1/GIP/glucagon	HM15211	Hanmi Pharmaceuticals	Phase I	[40,41,42]
	NN9423; NNC 92041706; Tri-agonist 1706	Novo Nordisk	Phase I	[67]

Abbreviations: GIP, Glucose-dependent insulinotropic peptide; GLP-1, Glucagon-like peptide-1; GLP-1RA, Glucagon-like peptide-1 receptor agonists; NA, Not available; LFT, Liver function tests; DPP-4, Dipeptidyl peptidase-4; PO, Oral administration; SC, Subcutaneous administration.

## Abbreviations

ALT	Alanine aminotransferase
AMPK	AMP-activated protein kinase
APRI	AST to platelet ratio index
AST	Aspartate aminotransferase
BA	Bile acids
BMI	Body mass index
CM	Chylomicron
CYP	Cytochrome P450
DCA	Deoxycholic acid
DIO	Diet-induced obesity
DPP-4	Dipeptidyl peptidase 4
FXR	Farnesoid X receptor
FGF	Fibroblast growth factor
FFA	Free fatty acid
GCDCA	Glycolchenodeoxycholic acid
GIP	Glucose-dependent insulinotropic peptide
GLP-1	Glucagon-like peptide-1
GLP-1RA	GLP-1 receptor agonists
GPCR	G-protein-coupled receptors
HbA1c	Glycosylated hemoglobin
HCC	Hepatocellular carcinoma
HFD	High-fat diet
IL	Interleukin
LCA	Lithocholic acid
LPS	Lipopolysaccharide
NAFLD	Non-alcoholic fatty liver disease
NASH	Non-alcoholic steatohepatitis
OCA	Obeticholic acid
PPAR	Peroxisome proliferator-activated receptor
RLP	Remnant lipoprotein
SCFA	Short-chain fatty acids
STAT3	Signal transducer and activator of transcription 3
T2D	Type-2 diabetes
TCDCA	Taurochenodeoxycholic acid
TG	Triglycerides
T2D	Type-2 diabetes
TLR	Toll-Like Receptors
TNF	Tumor necrosis factor
VLDL	Very low-density lipoprotein particles
ZO-1	Zonula occludens-1
